# Antiseptic technology: access, affordability, and acceptance.

**DOI:** 10.3201/eid0702.010216

**Published:** 2001

**Authors:** J M Boyce

**Affiliations:** Hospital of St. Raphael, New Haven, Connecticut, USA. JBoyce@srhs.org

## Abstract

Factors other than antimicrobial activity of soaps and antiseptic agents used for hand hygiene by health personnel play a role in compliance with recommendations. Hand hygiene products differ considerably in acceptance by hospital personnel. If switching from a nonmedicated soap to an antiseptic agent or increased use of an existing antiseptic agent for hand hygiene prevented a few more infections per year, additional expenditures for antiseptic agents would be offset by cost savings.


### Antiseptic Technology: Access, Affordability, and Acceptance

**John M. Boyce**
Hospital of St. Raphael, New Haven, Connecticut, USA

Factors other than antimicrobial activity of soaps and antiseptic agents used for hand hygiene by health personnel play a role in compliance with recommendations. Hand hygiene products differ considerably in acceptance by hospital personnel. If switching from a nonmedicated soap to an antiseptic agent or increased use of an existing antiseptic agent for hand hygiene prevented a few more infections per year, additional expenditures for antiseptic agents would be offset by cost savings.

Although the antimicrobial activity of preparations used by health-care workers for hand hygiene (soap and water or waterless antiseptic agents) is an important aspect of such preparations (1,2), other factors that influence the frequency of use of hand hygiene products by personnel are important.

### Access

The accessibility of sinks or other facilities may be an important factor, since nurses and other health-care personnel are expected to wash their hands frequently. Nurses wash their hands an average of 13 to 30 times each day, with as many as 44 times reported (Table 1) (3-5). In an observational study in an intensive care unit (ICU), nurses needed an average of 62 seconds to walk to a sink, wash and dry their hands, and return to the patient's bed (6). If nurses wash their hands for 10 seconds and 12 nurses work in an ICU, handwashing would require 16 hours of nursing time per shift (assuming 100% compliance with recommended handwashing practices). If nurses obtain an alcohol hand disinfectant from a bedside dispenser and 15 seconds is required for drying, 100% compliance would require 4 hours of nursing time per shift. Making a rapidly effective waterless antiseptic agent accessible at each patient's bedside should make it easier for nurses with heavy workloads to comply with recommended hand hygiene practices.

Few investigators have studied the relationship between access to sinks and handwashing frequency among health-care workers. Preston and colleagues (7) recorded personnel compliance with recommended handwashing in an open ICU with six beds and two sinks. After the ICU was converted into an isolation unit with 16 beds and 15 sinks (a sink for nearly every bed), the crude rate of compliance improved from 16% to 30%.

In an observational study in two ICUs, frequency of handwashing by health-care workers after contact with patients or their environment was recorded (8). In the medical ICU, where the sink:bed ratio was 1:1, personnel complied with recommended handwashing measures 76% of the time. In the surgical ICU, where the sink:bed ratio was 1:4, compliance decreased to 51%, indicating that improved access to handwashing facilities increases handwashing compliance. However, differences in handwashing compliance on medical and surgical services may be related to factors such as the number of opportunities for handwashing and attitudes of personnel toward hand hygiene (9).

Table 1. Frequency of handwashing per shift by health-care workers

| Author | Average/shift | Range |
|---|---|---|
| Ojajarvi (3) | 20-30 | 11-44 |
| Larson (4) | 16-25 | <8–25+ |
| Boyce (5) | 13-15 | 5–27 |

Address for correspondence: John M. Boyce, Hospital of Saint Raphael, 1450 Chapel Street, New Haven, CT 06511, USA; fax: 203-789-4239; e-mail: JBoyce@srhs.org

In a study of the impact of sink location on incidence of nosocomial infections (10), patients whose beds were located next to a sink had a 26% reduction in risk for infection compared with those whose beds were located farther away from a sink. In addition to placing sinks near patient beds whenever possible, hospitals should ensure that medical equipment adjacent to the patients' beds (e.g., ventilators or intravenous pumps) does not obstruct access to sinks. Physical barriers that restrict access to sinks may discourage personnel from washing their hands.

Automated handwashing machines have been tested, usually for improving the quality or the frequency of handwashing (11,12). Health-care personnel used these automated sinks infrequently, and they do not appear to be a useful solution to improving hand hygiene.

Other investigators observed health-care worker compliance with recommended hand hygiene practices in a medical ICU unit during three periods (13). During the baseline period, hands were washed with soap and water. Then, an alcohol-based hand disinfectant was made available, with one alcohol dispenser for every four beds. In the third period, additional dispensers were added so that there was one alcohol dispenser for each bed. During the baseline period, 25% of health-care workers washed their hands when recommended. Hand hygiene compliance improved to 41% when one alcohol dispenser was made available for every four beds and to 48% when a dispenser was placed next to every bed. This study also suggests that better access to hand hygiene facilities results in improved compliance.

### Cost

Few data are available regarding the cost of antiseptic agents used for hand hygiene. In 1999, a 450-bed community-teaching hospital spent $22,000 on 2% chlorhexidine-containing preparations, plain soap, and alcohol hand rinse, for a cost of $0.72 per patient per day (Figure 1). If hand hygiene supplies for clinics and non-patient care areas are included, the total annual budget for soaps and hand disinfectants was $30,000, or approximately $1 per patient per day. Because of different use patterns and varying product prices, annual hand hygiene budgets at other institutions could vary considerably.

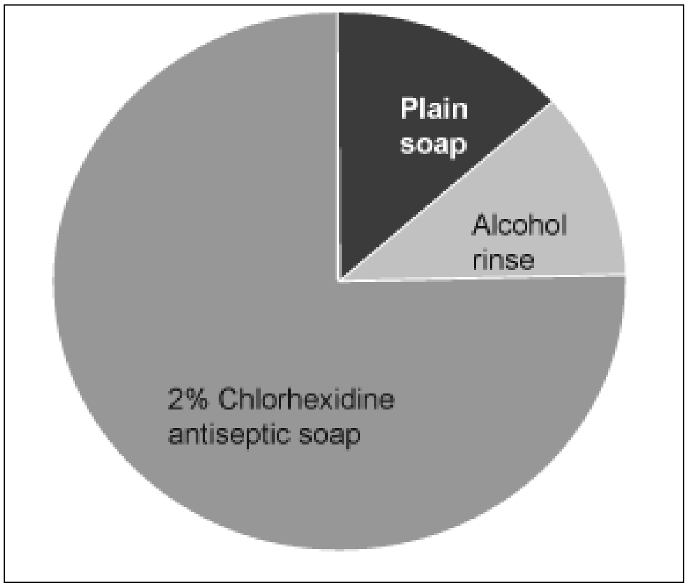

Figure 1. Annual expenditures for hand hygiene products used in patient care areas in a 450-bed community hospital, 1999.

The relative cost per liter was calculated for the products available through the hospital's buying group purchase contract (Table 2). The 2% chlorhexidine gluconate detergent was 1.7 times as expensive as the nonmedicated soap, and the alcohol-based hand gel was twice as expensive. Expenditures for soap or waterless hand disinfectants may be compared with excess hospital costs associated with nosocomial infections (Table 3). The excess hospital expense associated with four or five nosocomial infections of average severity is equal to the entire annual budget for soap and alcohol products used for hand hygiene in inpatient care areas. A single severe surgical site infection, lower respiratory infection, or bloodstream infection may cost the hospital more than the entire annual budget for antiseptic agents used for hand hygiene. If a change from nonmedicated soap to an antiseptic agent or a substantial increase in the use of antiseptic agents resulted in preventing a few additional nosocomial infections per year, the additional costs associated with using antiseptics would be offset by cost savings.

Table 2. Relative cost per liter of hand hygiene products

| Product category | Relative cost |
|---|---|
| Nonmedicated liquid soap | 1.0[a] |
| 2% chlorhexidine gluconate detergent | 1.7 |
| Alcohol-based hand gel A | 2.1 |
| Alcohol hand rinse | |
| A | 1.8 |
| B | 1.6 |
| Alcohol foam | |
| A | 4.7 |
| B | 4.8 |

[a]Nonmedicated liquid soap was arbitrarily assigned a relative cost of 1.0.

Table 3. Excess length of stay and hospital costs associated with nosocomial infections

| Site of infection | Increased length of stay (days) | Increased cost ($) Average | Maximum |
|---|---|---|---|
| Urinary tract | 1-4 | 600-930 | 8,280 |
| Surgical Site | 7-14 | 2,000-5,040 | 26,000 |
| Lower respiratory | 4-21 | 5,000-5,800 | 41,600 |
| Bloodstream | 4-24 | 3,000-40,000 | >40,000 |

Adapted from: Jarvis WR. Selected aspects of the socioeconomic impact of nosocomial infections: morbidity, mortality, cost, and prevention. Infect Control Hosp Epidemiol 1996;17:552-7.

### Acceptance

In studies of acceptance of hand hygiene products by health-care personnel, the adverse effects of frequent handwashing on the skin are considered an important issue by hospital personnel, one likely to affect the frequency of use of hand hygiene products (4,14). When hospital personnel rated five soap products for their tendency to cause skin dryness, cracking, or redness (3), the product that caused the greatest cracking and redness of the skin was least preferred by personnel. In a recent study (15), health-care workers subjectively evaluated four 4% chlorhexidine-containing products with respect to fragrance (smell), texture, lather, ease of rinsing, and tendency to cause itching. One of the four products evaluated was rated the worst in terms of smell, texture, and lather, but did not differ from the other preparations in ease of rinsing and tendency to cause itching. A subsequent questionnaire showed that the product with the undesirable smell and texture was the least popular among personnel.

Larson et al. (16) asked personnel to rate the condition of their skin before and after using water, bar soap, or one of three antiseptic preparations (antiseptics 1, 2, and 3). In self-assessments of skin condition, washing with bar soap or antiseptic 3 caused the most skin problems. In objective assessments of skin condition based on measurements of transepidermal water loss, handwashing with bar soap and antiseptic 3 produced the most skin damage. Clearly, not all handwashing preparations are equally acceptable to health-care personnel.

In the United States, health-care workers have believed that use of alcohol-based disinfectants causes excessive skin irritation and dryness. This attitude may be based on prior experience with products such as rubbing alcohol, which contains no emollients, or on outdated approaches to hand disinfection. Self-assessments of skin condition were recorded by volunteers who used an alcohol-based preparation without emollients and the same substance containing emollients (17). After 1 week of use and again after 2 weeks, the alcohol preparation containing emollients was thought to result in less damage to the skin.

In a recent prospective randomized trial (5), 29 nurses working on three hospital wards volunteered to participate. Half the nurses were randomly assigned to wash their hands with a nonmedicated soap (Soft N Sure, Steris, Inc., Mentor,

OH); the other half used an alcohol hand gel (Purell, GoJo Industries, Akron, OH) after patient contacts. Dispensers for the alcohol hand gel were placed outside each patient's room or in the patient's cubicle in the ICU. Nurses in both groups were asked not to use hand lotions or creams during the study period. After 2 weeks, all nurses resumed using standard soap-and-water hand washing and were allowed to use hand lotions or creams; the nurses who initially used soap and water switched to the alcohol hand gel regimen, and vice versa. Skin irritation and dryness were assessed by three methods: self-assessment by participating nurses, visual assessment by a study nurse, and electrical capacitance measurements of the skin on the dorsal surface of the nurses' hands (a measure of epidermal water content). Electrical capacitance measurements showed that nurses had more skin dryness if they washed their hands with soap and water than if they used the alcohol hand gel (Figure 2). Self-assessments by participants and visual assessments by the study nurse also showed that nurses had substantially greater skin irritation and dryness when using the soap-and-water regimen. On a questionnaire assessing attitudes toward the alcohol hand gel, 88% of nurses agreed or strongly agreed that the alcohol gel caused less dryness than soap-and-water handwashing; 92% agreed or strongly agreed that they would be willing to use the alcohol hand gel routinely. This study demonstrated that an alcohol hand gel containing appropriate emollients can achieve a high degree of acceptance by hospital personnel.

However, installing dispensers for alcohol-based hand disinfectants throughout a facility does not necessarily guarantee a high level of use. In a recent study, the number of liters of an alcohol hand disinfectant used per 1,000 patient-days increased substantially after implementation of a hospital-wide, multidisciplinary program to improve hand hygiene practices (18). The findings suggest that continuing educational and motivational efforts may be necessary for wide acceptance and frequent use of alcohol-based disinfectants by health-care workers.

### Conclusion

Ease of access to antiseptic agents and level of acceptance of products by personnel can influence compliance with recommended hand hygiene practices. Both these factors, as well as the costs and antimicrobial activity of preparations, should be taken into consideration in the selection of hand hygiene products for health-care workers.

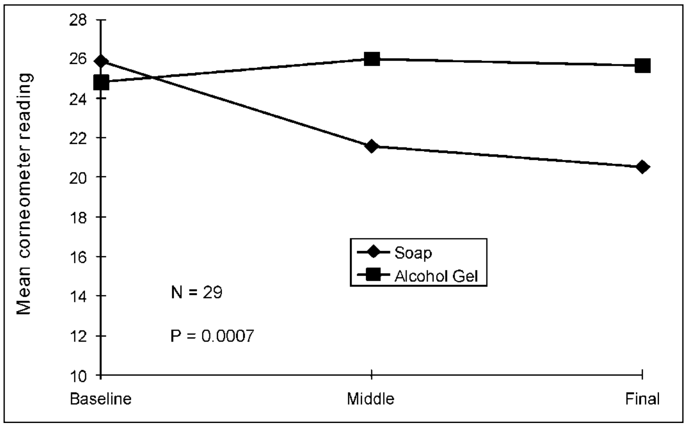

Figure 2. Electrical capacitance of dorsal hand skin surface (5).

Dr. Boyce is chief of the Division of Infectious Diseases at Hospital of Saint Raphael, New Haven, Connecticut. He is chair of a Hand Hygiene Task Force made up of members from the Hospital Infection Control Practices Advisory Comittee, the Society for Healthcare Epidemiology of America, the Association for Professionals in Infection Control and Epidemiology, and the Infectious Diseases Society of America. His research interests focus on prevention and control of multidrug-resistant nosocomial pathogens, including methicillin-resistant *Staphylococcus aureus* and vancomycin-resistant enterococci.

### References

1. Larson EL, APIC Guidelines Committee. APIC guideline for handwashing and hand antisepsis in health care settings. Am J Infect Control 1995;23:251-69.
2. Rotter M. Hand washing and hand disinfection. In: Mayhall CG, editor. Hospital epidemiology and infection control. 2nd ed. Philadelphia: Lippincott Williams & Wilkins; 1999. p. 1339-55.
3. Ojajarvi J. The importance of soap selection for routine hand hygiene in hospital. J Hyg (Camb) 1981;86:275-83.
4. Larson E, Killien M. Factors influencing handwashing behavior of patient care personnel. Am J Infect Control 1982;10:93-9.
5. Boyce JM, Kelliher S, Vallande N. Skin irritation and dryness associated with two hand-hygiene regimens: soap and water hand washing versus hand antisepsis with an alcoholic hand gel. Infect Control Hosp Epidemiol 2000;21:442-8.
6. Voss A, Widmer AF. No time for handwashing? Handwashing versus alcoholic rub: can we afford 100% compliance? Infect Control Hosp Epidemiol 1997;18:205-8.
7. Preston GA, Larson EL, Stamm WE. The effect of private isolation rooms on patient care practices, colonization and infection in an intensive care unit. Am J Med 1981;70:641-5.
8. Kaplan LM, McGuckin M. Increasing handwashing compliance with more accessible sinks. Infect Control 1986;7:408-10.
9. Pittet D, Mourouga P, Perneger TV, members of the Infection Control Program. Compliance with handwashing in a teaching hospital. Ann Intern Med 1999;130:126-30.
10. Freeman J. Prevention of nosocomial infections by location of sinks for hand washing adjacent to the bedside Abstract 60]. Program and Abstracts of the 33rd Interscience Conference on Antimicrobials and Chemotherapy. Washington, DC: American Society for Microbiology; 1993.
11. Larson E, McGeer A, Quraishi A, Krenzischek D, Parsons BJ, Holdford J, et al. Effect of an automated sink on handwashing practices and attitudes in high-risk units. Infect Control Hosp Epidemiol 1991;12:422-8.
12. Wurtz R, Moye G, Jovanovic B. Handwashing machines, handwashing compliance, and potential for cross-contamination. Am J Infect Control 1994;22:228-30.
13. Bischoff WE, Reynolds TM, Sessler CN, Edmond MB, Wenzel RP. Handwashing compliance by health care workers. Arch Intern Med 2000;160:1017-21.
14. Zimakoff J, Kjelsberg AB, Larsen SO, Holstein B. A multicenter questionnaire investigation of attitudes toward hand hygiene, assessed by the staff in fifteen hospitals in Denmark and Norway. Am J Infect Control 1992;20:58-64.
15. Scott D, Barnes A, Lister M, Arkell P. An evaluation of the user acceptability of chlorhexidine handwash formulations. J Hosp Infect 1991;18:51-5.
16. Larson E, Leyden JJ, McGinley KJ, Grove GL, Talbot GH. Physiologic and microbiologic changes in skin related to frequent handwashing. Infect Control 1986;7:59-63.
17. Rotter ML, Koller W, Neumann R. The influence of cosmetic additives on the acceptability of alcohol-based hand disinfectants. J Hosp Infect 1991;18 Suppl B:57-63.
18. Pittet D, Hugonnet S, Harbarth S, Mourouga P, Sauvan V, Touveneau S. Effectiveness of a hospital-wide programme to improve compliance with hand hygiene. Lancet 2000;356:1307-12.
